# *Candida albicans* Isolates from the Gut of Critically Ill Patients Respond to Phosphate Limitation by Expressing Filaments and a Lethal Phenotype

**DOI:** 10.1371/journal.pone.0030119

**Published:** 2012-01-13

**Authors:** Kathleen Romanowski, Alexander Zaborin, Vesta Valuckaite, Ronda J. Rolfes, Trissa Babrowski, Cindy Bethel, Andrea Olivas, Olga Zaborina, John C. Alverdy

**Affiliations:** 1 Department of Surgery, University of Chicago, Chicago, Illinois, United States of America; 2 Department of Biology, Georgetown University, Washington, D. C., United States of America; 3 Clinical Microbiology/Immunology Laboratories, University of Chicago, Chicago, Illinois, United States of America; New Jersey Medical School, University of Medicine and Dentistry of New Jersey, United States of America

## Abstract

*Candida albicans* is an opportunistic pathogen that proliferates in the intestinal tract of critically ill patients where it continues to be a major cause of infectious-related mortality. The precise cues that shift intestinal *C. albicans* from its ubiquitous indolent colonizing yeast form to an invasive and lethal filamentous form remain unknown. We have previously shown that severe phosphate depletion develops in the intestinal tract during extreme physiologic stress and plays a major role in shifting intestinal *Pseudomonas aeruginosa* to express a lethal phenotype via conserved phosphosensory-phosphoregulatory systems. Here we studied whether phosphate dependent virulence expression could be similarly demonstrated for *C. albicans. C. albicans* isolates from the stool of critically ill patients and laboratory prototype strains (SC5314, BWP17, SN152) were evaluated for morphotype transformation and lethality against *C. elegans* and mice during exposure to phosphate limitation. Isolates ICU1 and ICU12 were able to filament and kill *C. elegans* in a phosphate dependent manner. In a mouse model of intestinal phosphate depletion (30% hepatectomy), direct intestinal inoculation of *C. albicans* caused mortality that was prevented by oral phosphate supplementation. Prototype strains displayed limited responses to phosphate limitation; however, the *pho4*Δ mutant displayed extensive filamentation during low phosphate conditions compared to its isogenic parent strain SN152, suggesting that mutation in the transcriptional factor Pho4p may sensitize *C. albicans* to phosphate limitation. Extensive filamentation was also observed in strain ICU12 suggesting that this strain is also sensitized to phosphate limitation. Analysis of the sequence of *PHO4* in strain ICU12, its transcriptional response to phosphate limitation, and phosphatase assays confirmed that ICU12 demonstrates a profound response to phosphate limitation. The emergence of strains of *C. albicans* with marked responsiveness to phosphate limitation may represent a fitness adaptation to the complex and nutrient scarce environment typical of the gut of a critically ill patient.

## Introduction

Serious hospital infections leading to sepsis, organ failure, and death persist despite powerful antibiotics and strict environmental control measures. Pathogens that use the gastrointestinal tract reservoir as their primary site of colonization, such as *Pseudomonas aeruginosa* and *Candida albicans*, carry the highest case fatality rates when they disseminate and cause subsequent infection [1], [2], [3], [4], [5], [6], [7]. In addition there is increasing evidence that many patients enter the hospital as carriers of these pathogens in their stool as a result of chronic overuse of antibiotics [8], [9], [10], [11], [12]. Therefore there is a pressing need to understand the behavior of these pathogens from within the intestinal tract reservoir when patients are subjected to the physiologic and immune altering stresses of major surgery and extreme medical interventions.

While there is compelling evidence that disturbances in immune regulation and epithelial barrier function contribute to sepsis due to intestinal *C. albicans*, our laboratory has been interested in the local cues that shift intestinal microbes from indolent colonizers to lethal pathogens following extreme physiologic stress and injury. Our work has demonstrated that during surgical injury and other physiologic insults, compounds are released by host tissues that bind to and/or are taken up by the model opportunistic pathogen *P. aeruginosa* resulting in activation of its quorum sensing system leading to the expression of a lethal phenotype [13], [14], [15]. Once locally activated, microbes need not disseminate to cause sepsis or remote organ failure as they can employ a variety of virulence tactics that can perturb homeostasis and subvert clearance mechanisms [16]. In this clinical context, we hypothesized that local phosphate concentration at specific colonization niches could represent an important cue by which many pathogens evaluate the resources, health status, and hence suitability for colonization versus invasion in a given host [17], [18], [19], [20], [21]. We further hypothesized that once an extreme degree of phosphate depletion is reached, various microbes will respond by expressing a lethal phenotype.

*Candida albicans* is an eukaryotic opportunistic pathogen that resides on the mucosa of the gastrointestinal tract as well as the mouth, esophagus and vagina (reviewed in [22], [23]). Although this commensal organism normally colonizes mucosal surfaces in an asymptomatic manner, it can become one of the most significant causes of a disabling and lethal infection [24], [25], [26]. While the expression of virulence factors in *C. albicans* is described in response to certain environmental cues [22], [23], its phosphate-regulated virulence mechanisms are unknown. We hypothesized that *C. albicans* is signaled to express a virulent phenotype when it senses diminishing host resources as indicated by phosphate depletion. Therefore the aims of the present study were to determine the prevalence of *C. albicans* in the stool of critically ill patients and to determine the response of these strains to phosphate-limited conditions by assessing morphotype and virulence expression. To test this, we exposed *Candida* to low phosphate conditions, such as occur in the gut during stress, and observed them to become transformed to a more virulent state as judged by an increase in filaments known to be involved in invasion. Filament formation was associated with death in animals. Provision of phosphate as a countermeasure protected animals (worms and mice) against the lethal effect of *Candida albicans* by preventing its transformation to the filamentous form. *C. albicans* laboratory prototypic strains were included in the analysis for comparison and to help to define what role, if any, the transcription factors Pho4p and Grf10p play in the phosphate-mediated filamentation response.

Results demonstrated that *C. albicans* isolates from the stool of critically ill patients were transformed to a highly virulent and lethal phenotype during exposure to phosphate limitation. The response to phosphate limitation of prototypic strain SC5314 was minimal *in vivo* although its production of biofilm was significantly increased *in vitro* and its lethal effect in animals was attenuated by providing excess phosphate. Filamentation was highly inducible in the *C. albicans pho4*Δ during phosphate limitation compared to its isogenic parent strain SN152, suggesting that mutation in *PHO4* may sensitize *Candida* to phosphate depletion as a result of decreased expression of *PHO* genes. Similarly extreme filamentation in the ICU12 isolate and in the *pho4*Δ mutant suggested that ICU12 may carry a mutation in *PHO4* or an additional gene involved in its regulation. Sequencing *PHO4* from ICU12 although revealed five substitutions in the gene none indicated a loss of function. Interestingly, qRT-PCR of *PHO4* expression and secreted phosphatase activity demonstrated a robust response of ICU12 to phosphate limitation. These findings suggest that certain isolates of *C. albicans* may have a competitive advantage over other microbes for phosphate sources when faced with the nutrient poor conditions typical of the intestinal environment in critically ill patients.

## Results

### *Candida albicans* is a predominant microorganism in the stool of critically ill patients

**Figure 1:**
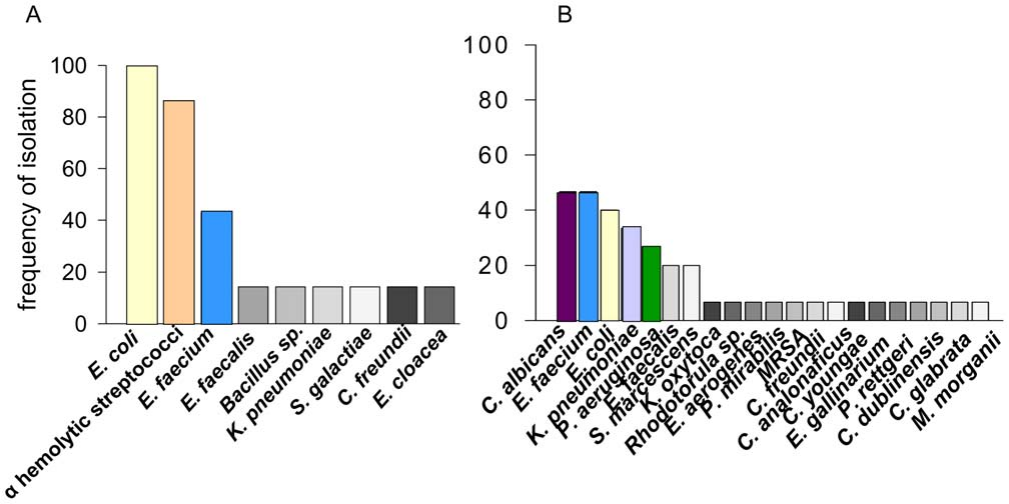
Frequency of cultured microbial isolates. (A) Organisms from the stool of 7 healthy human volunteers and (B) Organisms from the stool of 15 critically ill patients confined to a care unit (ICU). 100% frequency of isolation indicates that that all patients tested were positive for a given species. Color bars reflect the species isolated at a frequency > 20%, each species has an unique color, and grey-scale bars reflect the species isolated at a frequency of <20%.

Stool samples were collected from 15 critically ill patients and 7 healthy volunteers, and were cultured under aerobic conditions. Results revealed *Candida albicans*, *Enterococcus faecium*, *Escherichia coli*, *Klebsiella pneumoniae*, and *Pseudomonas aeruginosa* to be predominant microorganisms in the stool of critically ill patients (Fig. 1). *C. albicans* and *P. aeruginosa* were identified in the stool of critically ill patients but not in healthy volunteers (Fig. 1). Nearly 50% of all critically ill patients studied harbored *C. albicans* in their feces.

### Phosphate-dependent morphological changes in *C. albicans* strains isolated from the stool of critically ill patients compared to prototypic strains

**Figure 2:**
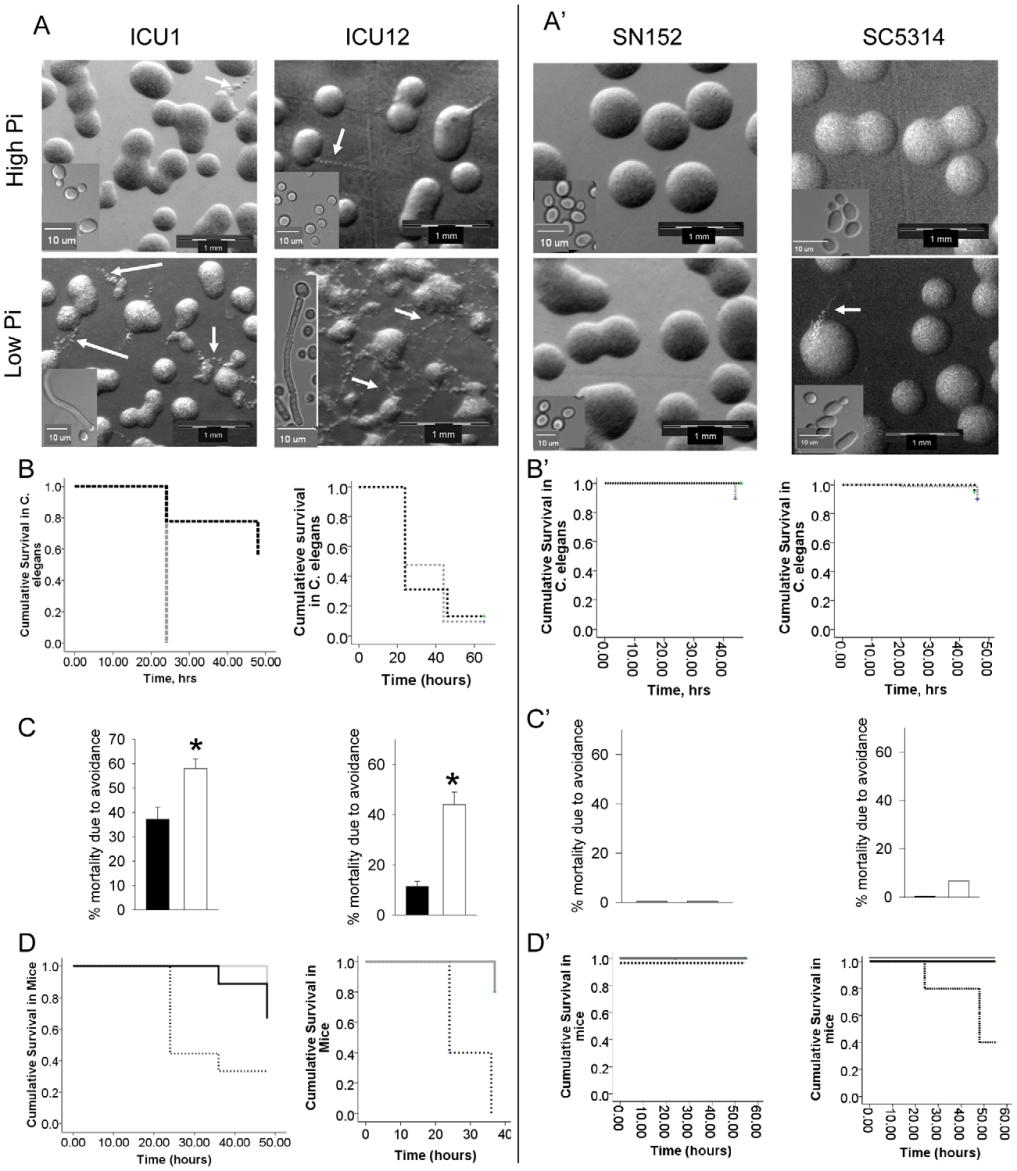
Phosphate-dependent filamentation and lethality for *C. albicans* strains isolated from the stool of critically ill patients (ICU) compared to prototypic strains. (A, A’) morphological changes in (A) ICU and (A’) prototypic strains on high (25 mM) and low (<0.1 mM) phosphate-supplemented solid PNMC media. Filaments are indicated by arrows. (B, B’) *C. elegans* killing assay. Kaplan-Meyer survival curves of *C. elegans* demonstrating the effect of phosphate supplementation on the lethal effect of *C. albicans*. Dark dotted line, PNMC, 25 mM Pi; light dotted line, PNMC, <0.1mM Pi. (C, C’) Mortality of *C. elegans* due to avoidance behavior of *C. albicans*. ▪ PNMC, 25 mM Pi; □ PNMC, <0.1mM Pi. n = 5 worms/plate, 7 plates/experiment, 2-3 independent experiments for each *C. albicans* strain. Significant differences in survival (B) of *C. elegans* were demonstrated for *C. albicans* ICU1 (p<0.01), significant differences in mortality due to avoidance behavior (C) were demonstrated for *C. albicans* ICU1 and ICU12 (*p<0.01). (D, D’) Mouse model. Kaplan-Meyer survival curves demonstrating the effect of phosphate supplementation on the lethal effect of (D) ICU and (D’) prototypic strains of *C. albicans*. Black solid line, control (sham operated) mice; light dotted line, mice subjected to 30% hepatectomy; light solid line, mice subjected to 30% hepatectomy and drinking phosphate solution. n = 10 mice/group. p<0.01 for strains ICU1, ICU12, and SC5314.

The effect of phosphate limitation on the morphotype of *C. albicans* from isolates was examined during growth on solid PNMC medium that represents a modified NGM medium that we have previously used to determine the effect of phosphate limitation on *P. aeruginosa* morphotype and expression of a lethal phenotype [17]. This medium was used either without phosphate supplementation (PNMC-Pi↓) or with the addition of 25 mM potassium phosphate buffer, pH 6.0 (PNMC-Pi↑). We observed that following overnight growth at 37°C, the morphology of multiple clinical isolates of *C. albicans* was significantly altered; primarily smooth round colonies were observed on high phosphate medium whereas filamentous colonies predominated in low phosphate medium. We chosen two strains, ICU1 and ICU12 based on an initial screen of morphotypes and their relative degree of filamentation (see supplemental figure S1). In Fig. 2A, images of the two representative strains are displayed. As seen on the higher magnification images of single cells, the majority of cells on high phosphate medium were in the budding yeast form whereas pseudohyphal and hyphal forms were observed on low phosphate medium (Fig. 2A, insert panels). The most striking transformation of the filamentous phenotype was observed in strain ICU12 which demonstrated close to 100% filamentation with the production of multiple filaments from each colony on PNMC-Pi↓ medium; this strain even produced filaments on PNMC-Pi↑ medium, although to a lesser degree. Based on the morphological response, strains ICU1 and ICU12 were selected for use in a small animal model of phosphate limitation developed in our laboratory (see below). We also compared the response to phosphate limitation using the clinically isolated strain SC5314 [27], and two prototypic laboratory strains derived from it, BWP17 [28] and SN152 [29], that carry mutations in transcription factors that mediate the response to phosphate limitation [30] (Ghosh, Metzger, Fonzi and Rolfes, manuscript in preparation). Interestingly, SC5314 and SN152 exhibited a weak filamentation response to low phosphate as compared to the strongest responding ICU isolates (Fig. 2A’).

### Effect of phosphate limitation on the lethality of *C. albicans* in *C. elegans* and mice

It is widely recognized that filamentation is associated with *C. albicans* virulence [22], [23]. In order to determine if the formation of filaments induced under low phosphate conditions correlated with a lethal phenotype, we performed *C. elegans* killing assays previously developed in our lab that mimic intestinal phosphate depletion [17]. Similar to our previous experimental design, we included a pre-starvation phase to evacuate the intestinal tube of worms of all previously ingested material. Synchronized L4-young adult worms were washed in water and transferred onto sterile solid PNMC-Pi↑ and PNMC-Pi↓ plates for 3 hrs followed by a second transfer onto the same medium seeded with *C. albicans.* In preliminary experiments, we observed the appearance of filaments at the edge of *C. albicans* lawns after 2–3 days of incubation on PNMC-Pi↑ medium, possibly reflecting a filamentation response to nutrient deprivation due to high colony density. Therefore, we switched to low density *C. albicans* plates similar to those seen on images in Fig. 2 to prevent rapid consumption of nutrients at zones of high cell density. Every 24 hrs, worms were transferred onto freshly prepared *C. albicans* plates, and mortality was followed for up to 50–60 hrs. Results demonstrated that mortality in worms was observed with strain ICU1 in a phosphate-dependent manner whereas strain ICU12 caused high worm mortality under both low and high phosphate conditions consistent with the relative higher degree of filamentation in this isolate (Fig. 2B). Death in worms was mainly attributable to abnormal distension of the intestine with the accumulation of fungi inside the intestinal tube; hyphae penetrating through the cuticle were also observed but were rare. Additionally, we observed differences in the behavior of worms in response to yeast grown on low versus high phosphate medium. Worms displayed an avoidance behavior against *C. albicans* growing on low phosphate medium, an effect which was not observed on high phosphate medium. The behavior to avoid *C. albicans* resulted in worms dying on the dry wall edges of the dishes; it should be noted that we did not consider these worms in the Kaplan-Meyer survival curves to avoid confounding the effects of phosphate depletion on *C. albicans* mortality. However, the behavior of the worms and the increase in their death on the dish wall was so obvious that we also plotted these data (Fig. 2C). Prototype strains of *C. albicans* were significantly less virulent in the *C. elegans* assay as seen by survival curves and avoidance behavior (Fig. 2B’, 2C’).

To validate the effect of phosphate limitation in a clinically relevant animal model, we subjected mice to a 30% hepatectomy and short term starvation (water only for 48 hrs), an otherwise recoverable surgical injury known to result in intestinal mucosal phosphate depletion [31]. At the time of hepatectomy, we injected *C. albicans* directly into the cecum and followed mice for sepsis and mortality. To determine the role of the local intestinal phosphate concentration on the lethal effect of *C. albicans* in this model, an additional group of operated mice were intestinally replenished with phosphate via oral supplementation and the *C. albicans* inoculum was suspended in 25 mM phosphate solution prior to intestinal injection. Results demonstrated that mice exposed to both hepatectomy and intestinal *C. albicans* developed signs of sepsis (lethargy, chromodacctyrhhea, ruffled fur) and had a significantly higher mortality when compared to sham operated mice intestinally inoculated with *C. albicans* (Fig. 2D). Replenishment of intestinal phosphate in operated mice completely suppressed *C. albicans* lethality (Fig. 2D). These results demonstrated that phosphate depletion is a critical determinant of *Candida* pathogenicity.

**Figure 3:**
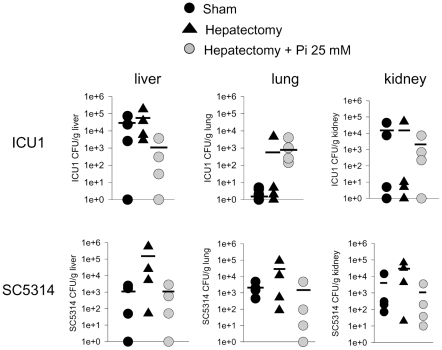
Dissemination patterns of *C. albicans* ICU1 and SC5314. Liver, kidney, and lung were isolated from surviving mice at 20 hrs following *C. albicans* cecal injection. Organs were weighed, homogenized, and 10 fold serial dilution in saline were plated on YPD and colony forming unit were determined. n = 4 mice/group.

Mortality was significantly less in mice injected with prototype strain SC5314 and was not observed in SN152 (Fig. 2D’). Despite the striking differences in mortality, the dissemination rate between *C. albicans* strains ICU1 and SC5314 in the sham-operated and hepatectomy mice was not significantly different (Fig. 3) and we detected no *Candida* cells in blood (data not shown). A trend toward less dissemination was noticed in hepatectomized mice drinking phosphate solution compared to those drinking water, however, it was not statistically significant. These observations indicate that the dissemination of *C. albicans*, as judged by organ culture, was not a critical determinant of mortality in this model.

**Figure 4:**
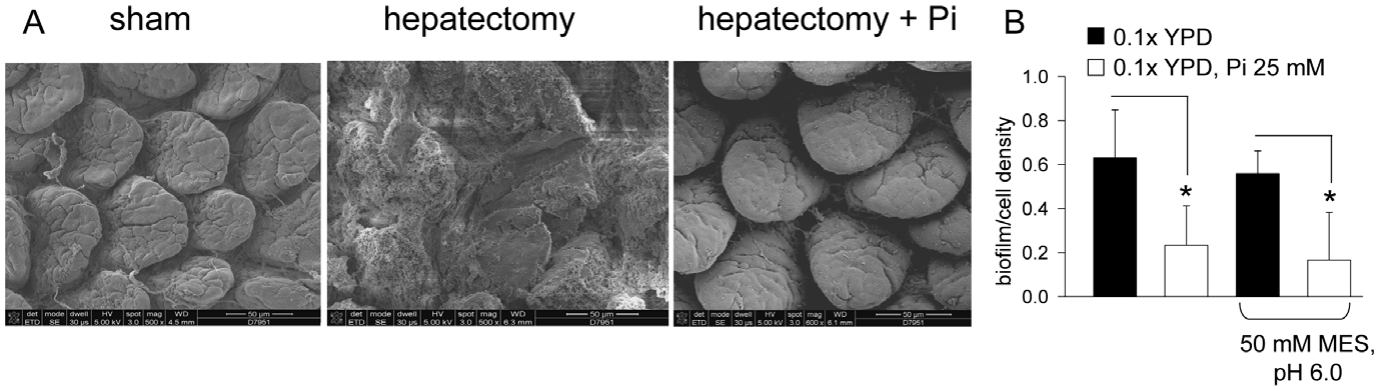
Phosphate attenuates the formation of biofilm in *C. albicans* SC5314 *in vivo* and *in vitro*. (A) Scanning electron microscopy (SEM) of intestinal tissues from mice with cecal injection of *C. albicans* SC5314. Intestinal segments were prepared as described in Materials and Methods and viewed in Fei Nova Nano SEM200 at a distance of 5 µm. Biofilm formation is seen on the intestinal mucosa of mice subjected to hepatectomy (center panel) but not in sham operated mice (left panel) and mice subjected to hepatectomy and drinking phosphate solution (right panel). (B) Biofilm formation for SC5314 at high and low phosphate concentration. Biofilm was evaluated using XTT/menadione method as described in Materials and Methods, and normalized to cell density. n = 6, *p<0.01.

We tested whether phosphate affected the ability to form a biofilm *in vivo* and *in vitro*. We performed a scanning electron microscopy (SEM) analysis of intestinal tissues from mice that had been intestinally injected with strains ICU1 and SC5314 following hepatectomy. For ICU1, we did not observe biofilm formation on the intestinal mucosa (data not shown). However, SC5214 formed a biofilm in the distal intestinal tract mucosa (illeum, cecum) but not in mice orally supplemented with phosphate or who underwent sham surgery (Fig. 4A). This finding raised the possibility that phosphate availability may suppress biofilm formation in SC5314. To test this, we measured biofilm formation in SC5314 grown in poor nutrient medium (0.1x YPD) and in the same medium supplemented with 25 mM phosphate buffer, pH 6.0 (Fig. 4B), with and without 50 mM MES buffer, pH 6.0. There was a significant increase in biofilm production in nutrient poor liquid medium that was suppressed by the addition of 25 mM Pi, independent of the MES buffer. Interestingly the virulent ICU1 strain was markedly attenuated in its ability to form a biofilm *in vitro;* however, despite the attenuation of biofilm formation in ICU1, biofilm production was still inhibited in high phosphate medium (data not shown).

### *GRF10* and *PHO4*-dependent morphological changes and lethality in *C. albicans* prototypic strains BWP17 and SN152

**Figure 5:**
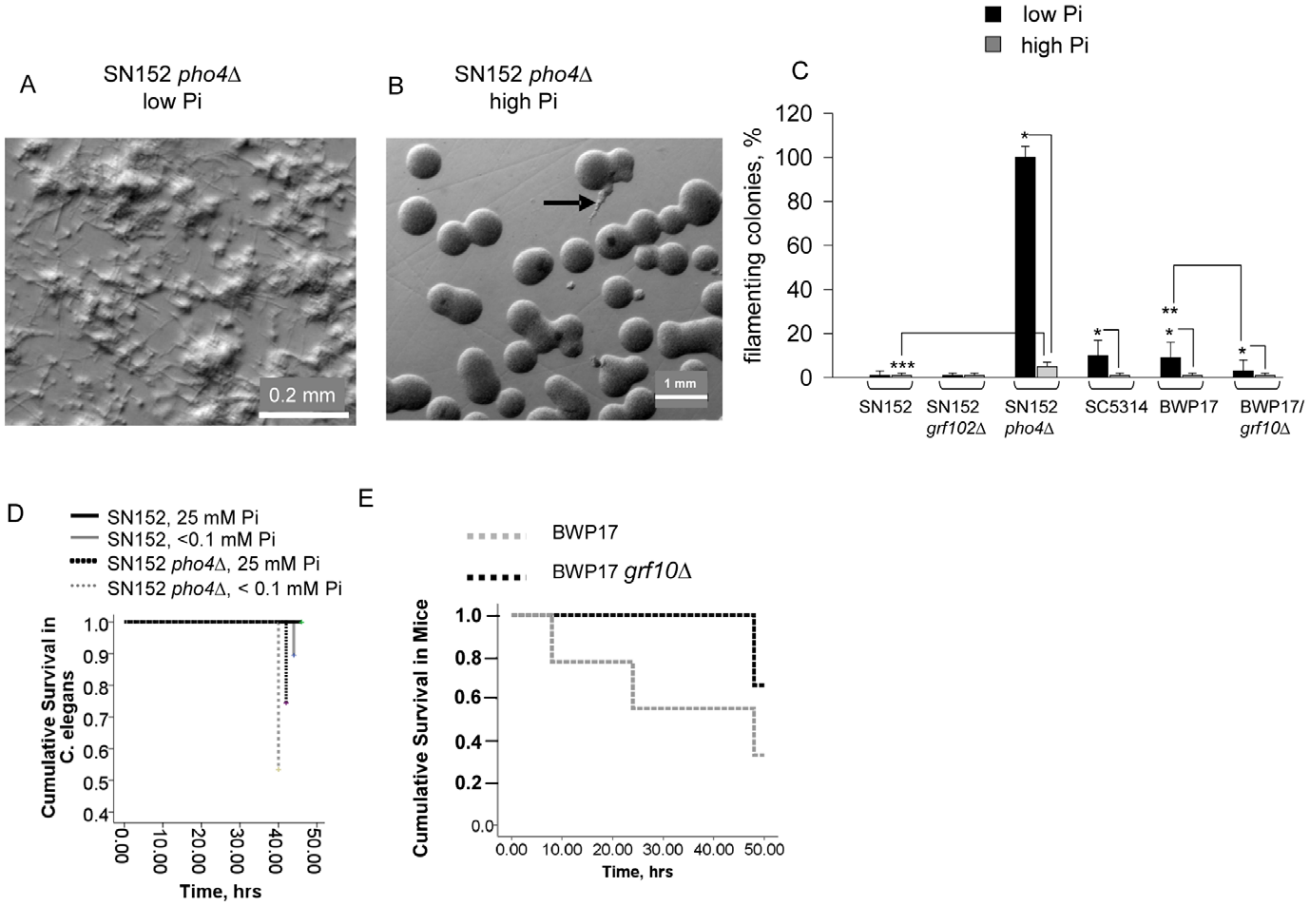
The effect of mutation in *CaGRF10* and *CaPHO4 on* the morphology and lethal effect of prototypic strains BWP17 and SN152. (A,B) Abundant filamentation is produced by *pho4*Δ on low phosphate solid medium (A) but not on high phosphate (B). (C) The percentage of filamented colonies in prototypic strains and its mutants *grf10*Δ and *pho4*Δ. n = 10 microscopy fields with 20-100 colonies/field, The number of filamented colonies on low Pi medium was significantly higher in SC5314, BWP17, and *pho4*Δ, *p<0.01, and in BWP17 low Pi as compared to *grf10*Δ low Pi, **p<0.01. On high phosphate medium, the number of filaments was significantly higher in *pho4*Δ compared to SN152, ***p<0.01. (D) Kaplan-Meyer survival curves in *C. elegans*. n = 70/variant, p<0.01 in between *pho4*Δ and SN152 induced mortality in low Pi medium. (E) Kaplan-Meyer survival curves in mice subjected hepatectomy and cecal injection of BWP17 or its derivative *grf10*Δ. n = 10/variant, *p<0.01.

The molecular details of yeast responsiveness to phosphate limitation rely mainly on studies in *S. cerevisiae* [32], [33] and recent work in *C. glabrata* [34]. There is a scarcity of data regarding *C. albicans'* response to phosphate, and therefore the potential roles of the proteins Pho4p and Grf10p, homologous to ScPho4p and ScPho2p, respectively, remain unknown. In addition there is no information regarding the effect of phosphate limitation on hyphae and pseudohyphae production. Therefore, we performed studies using *C. albicans* prototypic strains carrying mutations in *PHO4* and *GRF10.* First, we observed variation in the response to low phosphate among SC5314 and two laboratory strains, SN152 and BWP17 that are derived from it. SC5314 and BWP17 exhibited a low filamentation response to phosphate limitation and SN152 had virtually no filamentation response (Fig. 5C). Unexpectedly, we found that the *pho4*Δ mutant (in the SN152 strain background) produced an extensive amount of filaments on solid PNMC-Pi↓ (Fig. 5A), with filaments even appearing on high phosphate medium (Fig. 5B). The quantity of filaments produced on high phosphate medium by *pho4*Δ was significantly higher than that produced by the isogenic parent strain on low phosphate medium (Fig. 5C). The *pho4*Δ mutant was also more virulent than its parent SN152 in *C. elegans* killing assays (Fig. 5D). Conversely, the *grf10*Δ mutant (in the BWP17 strain background) was found to be attenuated in filamentation on low Pi (Fig. 5C) and demonstrated attenuated lethality in the mouse model (Fig. 5E). Together, these results suggest that mutation in the transcriptional factor Pho4p may sensitize *C. albicans* to phosphate limitation whereas we saw no obvious role for Grf10p in the phosphate response.

### Sequence of *PHO4* in *C. albicans* ICU12

Because of the similar responses of the ICU and *pho4*Δ strains to phosphate limitation, we hypothesized that the *PHO4* gene in the ICU isolates may have acquired mutations. To examine this, we amplified and sequenced *PHO4* from ICU12. Sequence analysis demonstrated several single nucleotide changes such as A90G, A149G, C476G, C942T, and T1711C as compared to SC5314 reference sequence. The A149G substitution led to the amino acid replacement of Asn59 with Asp, and the C476G substitution to replacement of Ser159 with Cys. We have also detected heterozygocity in the *PHO4* alleles, such as 429G/429A, 1122C/1122T, 1131G/1131T, 1176C/1176T, and 1815G/1815A. None of the amino acid changes affected the helix-loop-helix domain known to be involved in dimerization and DNA binding in transcriptional regulators. We therefore concluded that the extensive filamentation observed in strain ICU12 in response to phosphate limitation was not due to loss-of-function mutations in *PHO4*.

### Transcriptional response of *PHO4* to phosphate limitation

**Figure 6:**
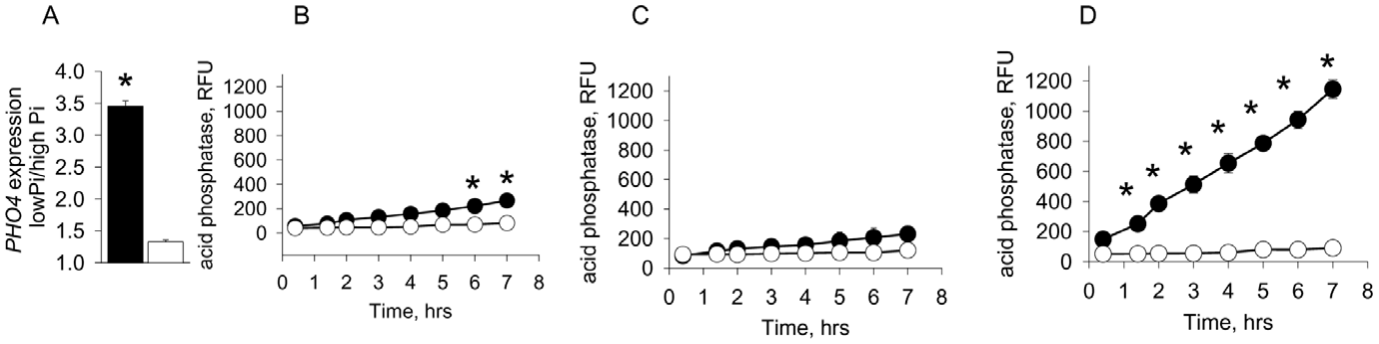
Expression of *PHO4* and production of acid phosphatase in response to phosphate limitation. (A) QRT-PCR analysis demonstrating the fold expression of *PHO4* in (▪) ICU12 and (□) SN152 growing on low phosphate vs high phosphate solid PNMC medium. n = 3, p<0.01. (B-D) Acid phosphatase activity in (B) SN152, (C) *pho4*Δ, (D) ICU12 grown in (•) liquid PNMC, 25 µM Pi and in (○) liquid PNMC, 25 mM Pi. n = 3, *p<0.01.

We wondered if the expression of *PHO4* could account for the phosphate response that we saw. Using our DNA sequence analysis, we designed primers to the conserved portions of *PHO4* and performed qRT-PCR analysis of the transcriptional response to phosphate limitation. Total RNA was isolated from strains ICU12, SN152, and *pho4*Δ that had been grown for 4 hrs on solid PNMC-Pi↑ and PNMC-Pi↓ media. The integrity analysis of RNA was examined with the Eukaryote Total RNA Nano Assay of the Agilent Bioanalyzer and demonstrated a ratio of 28S:18S∼ 2.0 and RNA integrity number (RIN) ∼ 9-10. Interestingly, we found a 3.5-fold increase in *PHO4* expression in strain ICU12 but only a 1.25-fold increase in SN125 in response to phosphate limitation (Fig. 6A). As expected, there was no qRT-PCR signal in the *pho4*Δ. This result shows that expression of *PHO4* can be altered by phosphate availability in some *Candida* isolates.

### Comparative analysis of phosphatase activity in strains SN152, *pho4*Δ, and ICU12

Given that Pho4p in *S. cerevisiae* transcriptionally activates the PHO regulon including *PHO5*, the major secreted acid phosphatase responsible for scavenging of phosphate, we decided to measure acid phosphatase activity in 3 strains of *C. albicans*: ICU12, SN152, and *pho4*Δ**.** We found that acid phosphatase activity was slightly increased in SN152, did not increase in *pho4*Δ but was dramatically increased in ICU12 (Fig. 6B). Taken together these findings demonstrate that *C. albicans* ICU12 responds to phosphate limitation by upregulating *PHO4* and secreted acid phosphatase expression.

## Discussion

The local environmental cues that shift microbes colonizing the intestinal tract of critically ill humans into a pathogenic state is a relatively unexplored area of investigation. Critically ill patients are exposed to a variety of physiologic stresses and medical therapies such as vasoactive drugs, high dose opioids, acid suppressing agents, and artificial nutrition consisting of either highly processed food that is absorbed proximally in small intestine or intravenous feeding. In the aggregate, these factors are likely to significantly shift the local intestinal microenvironment leading to changes in the composition and character of the colonizing flora as microbial communities adapt to the chaos of an ever changing environment. In addition, critically ill patients invariably receive long courses of antibiotics even when no infectious agent is identified, promoting the growth of multiple opportunistic pathogens including *Candida* species [24], [26], [35]. In our study, *Candida albicans* was highly prevalent in the stool of critically ill patients who were exposed to conditions known to deplete phosphate such as intravenous nutrition and various physiologic insults including major surgery [35]. Importantly, among these *Candida* isolates, several demonstrated a significant response to phosphate limitation *in vitro* expressing a filamentous and lethal phenotype.

We used two previously described animal models (*C. elegans*, mice) in which we previously established conditions of phosphate depletion [17], [31]. Both models were introduced for the first time to examine *C. albicans* phosphate-related lethality. Our mouse model included physiological stress (starvation and 30% hepatectomy) coupled with direct injection of *C. albicans* into the distal intestine. Direct inoculation to the site where microbes most commonly colonize and cause invasion (i.e. distal intestine) is more representative of the clinical scenario when gut microflora cause sepsis. The approach of oral inoculation in the drinking water or via gavaging [36], [37] is confounded by pH and other factors and generally does not cause sepsis in the absence of immunosuppression and antibiotics. Mortality in *C. elegans* was consistently associated with filamentation, although several technical aspects were noticed that affected the applicability of this model: 1). The optimal temperature for maintenance of *C. elegans* (25°C) was below the optimum for filamentation of *C. albicans* (37°C) as we observed loss of hyphal development when the *C. albicans* plates were maintained at 25°C; 2). *C. albicans* grew faster on PNMC-Pi↑ plates leading to a subsequent higher level of cell accumulation in the intestinal tube; 3). The mortality of worms on the walls of the dishes due to avoidance behavior of *C. albicans* under phosphate limited conditions artificially decreased the amount of worms counted as dead in survival plots. Nonetheless, our mouse model validated our *C. elegans* data confirming phosphate-dependent filamentation and lethality for *C. albicans* strains isolates obtained from the gut of critically ill patients. Importantly, our mouse model demonstrates that lethal gut-derived sepsis due to *C. albicans* can occur without exposing mice to immunosuppressant or antibiotics which have been previously reported to be required for *C. albicans*-related mortality [38].

There is very limited information on the molecular responsiveness of *C. albicans* to low phosphate. Indirect evidence has been presented by Cassone et al. [39] who observed a reduction of phosphate containing compounds in hyphae as well as hyphal growth appearance in the absence of external Pi. More detailed studies on phosphate regulation have been performed in *Saccharomyces cerevisiae* [32], [33] and more recently in *Candida glabrata* [34]. Genes in the *PHO* regulon encode acid and alkaline phosphatases and high affinity transporters that facilitate phosphate scavenging and uptake, and these genes are transcribed in response to phosphate concentration. A critical step in this response is the phosphorylation status of the transcriptional factor Pho4p which regulates its nuclear localization. When dephosphorylated under phosphate limited conditions, Pho4p localizes in the nucleus; in *Saccharomyces,* it forms a complex with its co-activator ScPho2p that leads to *PHO* regulon transcriptional activation [32], [40] whereas in *C. glabrata*, CgPho4p activity is independent of CgPho2p [34]. In *C. albicans*, the CaPho4p transcriptional regulator is required for growth in phosphate-depleted medium while CaGrf10p, the homologue to ScPho2p, is not required for the response to phosphate limitation [30] [Ghosh, Metzger, Fonzi and Rolfes, manuscript in preparation]. Yet the precise roles of Pho4p and Grf10p in *C. albicans* virulence remain unknown. In the current study, the observation of extensive filamentation in the *C. albicans pho4*Δ indicates the importance of the *PHO* genes that are involved in phosphate uptake and utilization. It is possible that the *pho4*Δ mutant is sensitized to phosphate depletion due to lowered expression of *PHO* genes and is therefore hyperfilamentous as a consequence. The similarity in the abundance of filamentation inICU12 and *pho4*Δ suggested that the clinical isolate ICU12 carries a mutation in *PHO4* or additional genes involved in its regulation. However, analysis of the *PHO4* sequence in this strain revealed only small differences compared to SC5314 and no mutation in the helix-loop-helix domain. The qRT-PCR analysis demonstrated a 3.5 increased expression of *PHO4* in ICU12 under phosphate limitation that correlated to increased acid phosphatase activity.

Phosphatase activity in *C. albicans* has been previously demonstrated to be very low (Ghosh, Metzger, Fonzi and Rolfes, manuscript in preparation) or delayed [41]. In the current work, we found that the phosphate limitation had a dramatic effect on phosphatase activity in strain ICU12. The ability to sequester phosphate undoubtly benefits fungi to compete with neighboring microflora for phosphate resources. The extensive filamentation observed in ICU12 during phosphate limitation could represent a mechanism by which it invades tissues to obtain phosphate. Although both the *pho4*Δ mutant and ICU12 responded to phosphate limitation with increased filamentation, ostensibly to obtain phosphate from host tissues, the former strain is incapable of a regulated response to phosphate limitation since it lacks the required transcriptional regulator Pho4p. *C. albicans* ICU12 up-regulated Pho4p in response to phosphate limitation, and as such, increased the expression of the secreted phosphatase facilitating the access of phosphate from host stores. Taken together these results suggest that The contrast between SN152 and ICU12 beg a more detailed genetic understanding of the mechanisms that lead to ICU12 strain's hypersensitivity to phosphate. Interestingly, we have previously demonstrated a similar extreme response to phosphate limitation among multi-drug resistant strains of *P. aeruginosa* isolated from critically ill patients [42], [43]. These strains responded to phosphate limitation with activation of high affinity phosphate binding proteins belonging to the DING/PstS family that formed outer surface appendages presumably for phosphate scavenging. We ascribe the altered behavior of ICU strains of *C. albicans* to their evolvability in the complex environment of a critically ill human. Modern treatment strategies expose patients to prolonged and broad-spectrum antibiotics, extreme life-saving measures, and intravenous nutrient delivery. In the aggregate exposure to this complex ecology selects for *P. aeruginosa* and *C. albicans* strains that express phenotypes that may not be observed otherwise. Perhaps the emergence of *C. albicans* expressing a highly virulent response to phosphate limitation represents a fitness adaptation to this harsh environment.

The differential involvement of the Pho2p and its homologues in *S. cerevisiae, C. glabrata* and *C. albicans* indicates differences in their evolutionary histories and ecological niches. This transcription factor from the human pathogenic *Candida* species (CgPho2p and CaGrf10p) is not required for growth in the absence of phosphate; instead we and others have shown that the *grf10*Δ mutants are defective in filamentation [30](Ghosh, Metzger, Fonzi and Rolfes, manuscript in preparation). In *S. cerevisiae*, ScPho2p (the CaGrf10p homologue) is involved in the regulation of a diverse array of genes with three coregulators and pleiotropic effects [40], [44]. It therefore may be possible that in *C. albicans,* Pho4p is involved in phosphate-dependent filamentation independent of Grf10p while Grf10p can interact with other transcriptional regulators.

In summary, data from the present study provide compelling evidence that various isolates of the eukaryotic pathogen *C. albicans* can respond to phosphate limitation with enhanced virulence resulting in host death. Further work will be necessary to elucidate the molecular mechanisms of this response and will require deep sequencing of our clinical isolates, assessment of their genome wide transcriptional responses to varying concentrations of phosphate, and the generation of an appropriate library of mutants.

## Materials and Methods

### Microorganisms

Microorganisms were cultured from stool samples of critically ill patients using selective media Macconkey II, TSA II 5% SB, Columbia CAN 5% SB, and Pseudomonas isolation agar (PIA). Most isolates identified as *Pseudomonas aeruginosa* were oxidase positive, oxidized glucose, hydrolyzed arginine, and grew at 42°C. Remaining *Pseudomonas aeruginosa* and all other gram negative bacilli were identified by the Vitek 2 system (bioMerieux, Inc. Durham, NC). Susceptibility testing of gram negative *Bacilli* was performed by the Vitek 2 system or by disk diffusion. Gram positive *Cocci* were identified by standard manual methods. Enterococcus speciation was performed by the Vitek 2 system. Susceptibility testing of MRSA and *Enterococci* was performed by the Vitek 2 system. Susceptibility testing of the other gram positive *Cocci* was performed using a combination of disk diffusion and E-test strips. A positive germ tube test identified *Candida albicans*. Other yeasts were identified using a variety of standard methods. Susceptibility testing of *Candida* was performed using the Sensititre YeastOne® MIC panel (TREK Diagnostic Systems Inc., Cleveland, OH).

All clinical *C. albicans* strains were named ICU# with the number given corresponding to the consecutive patient number. *C. albicans* strains SC5314 [45], BWP17 [28] and its derivative mutant *grf10*Δ (Ghosh et al; manuscript in prep), SN152 [29] and its derivative mutants *grf10*Δ and *pho4*Δ [30] were used as prototypic strains. PNMC medium contained 2.5 g/L peptone, 3 g/L NaCl, 1 mM MgSO_4_,1 mM CaCl_2_ and 17 g/L agar (Fisher). It was used without phosphate supplementation (PNMC-Pi↓ medium) or with the addition of 25 mM potassium phosphate buffer, pH 6.0 (PNMC-Pi↑).

### Human subjects

Human fecal samples were obtained from consecutive patients hospitalized at the University of Chicago Medical Center Care Units (ICU's) and human healthy volunteers of > 25 years old with no history of antibiotic treatment for 12 months prior to sampling. Patients hospitalized in the various surgical ICU's (cardiac, transplant, general surgical, burn units) were approached and consented to participate in the study. The written informed consent was provided by study participants and/or their legal guardians. Protocol #1646B approved by the University of Chicago Institutional Review Board was followed during stool sample collection. We confirm that the University of Chicago Institutional Review Board specifically approved this study.

### Small animal models

#### Nematode *C. elegans* model of phosphate limitation

*Caenorhabditis elegans* strain N2 was used in all experiments. *C. elegans* maintenance was performed accordingly to the “Maintenance of *C. elegans*” (http://www.wormbook.org/chapters/www_strainmaintain/strainmaintain.html). Synchronization of nematodes was performed as follows: 50–70 adult worms were transferred from stock plates onto sterile agarized NGM plates and allowed to lay eggs for 3 hrs at room temperature. No additional bacteria as the food source were added due to the fact that nematodes can auto-seed the plate with *E. coli* OP50 remaining in their digestive tubes. After 3 hrs, all adult nematodes have been removed from plate, while eggs were allowed to hatch and larvae allowed growing up to the L4-young adult stage. For pre-fasting, nematodes were further transferred onto plain agarized plates. In 3 hrs, worms were re-transferred to experimental *C. albicans* plates prepared at the same low colony density as for morphological examination. The low colony density was chosen since we noticed the formation of filaments in local spots with high colony density even at 25 mM Pi perhaps due to local consumption of nutrients. To prepare the plates, *C. albicans* cells were harvested from solid YPD (1%, w/v, yeast extract, 2%, w/v, peptone, 2%, w/v, dextrose, 1.5% agar) medium grown overnight at 37°C, and suspended in either water or 25 mM potassium phosphate buffer, pH 6.0 to OD600 of 0.1-0.2. 25 µl of solutions was spread on (PNMC-Pi↓) or (PNMC-Pi↑) agarized media, respectively. Plates were incubated overnight at 37°C, adjusted to room temperature for 1 hr, seeded with 5 pre-starved worms in 7 replicates per experiment performed, and incubated at 23°C. 2-3 independent experiments were performed with each *C. albicans* strain.

#### Mouse model of lethal gut-derived sepsis

**.**All experiments were approved by the Animal Care and Use Committee at the University of Chicago (IACUC protocol 71744). All studies involving mice conformed to the Animal Welfare Act and NIH Guidelines for the care and use of animals in biomedical research and with the University of Chicago Carlson Veterinary guidelines. Mice were housed in the animal facility at the University of Chicago. This facility has all the necessary personnel (veterinarians and support staff) and experience to handle the animals in accordance with Federal Regulations. All live infections in mice were performed in a class II biosafety cabinet in the biohazard facility. The method of euthanasia was consistent with the recommendations of the Panel on Euthanasia of the American Veterinary Medical Association and received approval by the University of Chicago IACUC. Every effort to avoid discomfort, distress, pain and injury was made in accordance with the conduct of scientifically sound research. Male C57BL6/HSD mice weighing 18 to 22 g were used for all experiments. Experimental protocol: Mice were routinely fed tap water and Harland Teklad feed. 16–18 hours prior to initiation of the experiment mice were fasted and allowed access to either tap water, or 25 mM potassium phosphate buffer (PB) pH 6.0. A bloodless 30% hepatectomy was performed using aseptic technique through a midline incision as previously described [46] followed by the injection into the cecum via direct puncture of 1×10^7^ *C. albicans* cells in 200 µl of either water or 25 mM potassium phosphate buffer, pH 6.0. Animals injected with *C. albicans* suspended in water were allowed to drink water, and animals injected with *C. albicans* suspended in phosphate solution were allowed to drink phosphate solution ad libitum only for the remainder of the study period. In the sham-operated group, mice underwent a laparotomy but no hepatectomy and underwent direct cecum injection of *C. albicans* suspended in water. Mice were followed for 48 hours for the development of signs of sepsis and mortality. Control groups included hepatectomy operation without injection of *C. albicans* that as we have previously described [46], [47] and verified in present study does not cause mice mortality. n = 5 mice/group/experiment in 2-3 independent experiments with each *C. albicans* strain.

### Dissemination analysis

Blood, liver, kidney, and lung were isolated from surviving mice at 20 hrs following *C. albicans* cecal injection. Organs were weighed, homogenized, and subjected to 10 fold serial dilution in saline and then plated on YPD plates, and *C. albicans* colonies counted at 48 hrs, n = 4 mice/group. 50 µl of blood was also plated and quantitative counts evaluated at 48 hours.

### Colony morphology

*C. albicans* colony morphology was examined using Olympus SZX16 stereomicroscope. For each plate the colonies were counted and percent filamentation was determined. The experiments were reproduced at least 3 times. The cell phenotype was examined using the Axiovert100TV Scope (Zeiss) microscope with a 63X oil objective.

### Biofilm production

Biofilm formation was assessed using a protocol adapted from Pierce and colleagues [48]. *C. albicans* was grown on Yeast Peptone Dextrose (YPD) agar plates overnight at 37°C. A single colony from the plate was placed in liquid YPD broth and placed on a shaker overnight at 37°C. The 1∶100 dilution was made in 0.1xYPD media with or without addition of 25 mM phosphate buffer, pH 6.0. 50 mM MES pH 6.0 was included in media when needed. In each well of a 96 well plate, 200 µl of sample was added. Plates were allowed to incubate statically at 37°C overnight, and cell density was measured by absorbance at 600 nm. Then medium was carefully aspirated so as to not disrupt the biofilm and wells were washed 3 times with sterile PBS to remove non-adherent cells. XTT/menadione was added to each well and allowed to incubate at 37°C for 3 hours. The absorbance at 490 nm was read on Bio-TEK PowerWave XSTM Microplate Scanning spectrophotometer and normalized to the absorbance at 600 nm.

### Scanning electron microscopy (SEM)

Intestinal segments were placed on ice cold PBS, transferred to 4% paraformaldehyde Solution (USB 19943) and kept in EtOH-PBS solutions for 40 minutes per step (25% EtOH-PBS, 50% EtOH-PBS, 75% EtOH-PBS, 90% EtOH-PBS, 100% EtOH 2X accordingly) . The samples were then transferred to 50% EtOH-HMDS (Hexamethyldisilazane Ted Pella 18605) and 100% HMDS and kept for 1 hour each step. Finally samples were left in 100% HMDS overnight in the hood to ensure evaporation. Next the samples were affixed very carefully to carbon stubs (Ted Pella 16111-9, Specimen mounts, Aluminium, 9 mm high, Ted Pella Carbon tape 9 mm, 16084-3), and sputter coated with 80%Pt/20%Pd to 12 nm with Cressington Sputter Coater 208HR. The samples were viewed in Fei Nova Nano SEM200 at a distance of 5 µm.

### *PHO4* sequencing in *C. albicans* ICU12

Several PCR products covering the entire *PHO4* were sequenced using an Applied Biosystems 3730XL 96-capillary sequencer at the University of Chicago Cancer Research Center DNA Sequencing Facility. Nucleotide and amino acid sequences are deposited in GenBank (accession number BankIt1493375 SeqPHO4 JQ023667).

### QRT-PCR analysis

*C. albicans* cells were plated on (PNMC-Pi↓) or (PNMC-Pi↑ agarized media as described in Materials and Methods section “Nematode *C. elegans* model of phosphate limitation” and grown for 4 hrs at 37°C. 1.5 ml of RNA stabilization reagent RNAlater (Ambion) was poured onto the plate, and cells were gently scraped from the dish and centrifuged at 12,000xg, 5 min, 4°C. Supernatant was removed, and RNA was isolated from the cell pellet using RiboPure™-Yeast kit (Ambion, Inc.) followed by treatment with DNA-free kit (Ambion, Inc.). The RNA integrity was examined with Eukaryote Total RNA Nano Assay using Agilent 2100 Bioanalyzer (Agilent Technologies, Inc.) and demonstrated a ratio of 28S:18S∼ 2.0 and RNA integrity number (RIN) ∼ 9-10. The purity/concentration of RNA was determined using a NanoDrop 1000 (Thermo Scientific).The cDNA synthesis from 1 µg total RNA was performed using the high capacity RNA-to-cDNA kit (Applied Biosystems), and 1 µl of 1∶50 diluted cDNA was used in the qRT-PCR analysis in the total reaction mixture of 10 µl containing 5 µl of Platinum SYBR Green qPCR SuperMix-UDG with ROX (Invitrogen) and 0.2 µl of 10 µM each primers. qRT-PCR was performed using 7900HT Fast Real-Time PCR System (Applied Biosystems). The program for amplification had an initial heat step at 50°C for 2 min, followed by the denaturation step at 95°C for 15 sec, and then followed by 40 cycles of 95°C for 15 s and 60°C for 1 min. The specificity of the reaction was monitored by melt-curve analysis following the real-time program. The gene expression was normalized to *TDH3* [49], [50]. Fold change was determined using normalized expression in PNMC-Pi↑ as 100%. The Primer3 software was used to design primers for qRT-PCR *PHO4*-223-F 5′ CAAAACACGCCACATATTGTTT 3′, and *PHO4*-339-R 5′ GCCTGCAGACTGGTTAGTGT 3′. The primers were designed as *TDH3*-54-F 5′ AAGAGTTGCTTTGGGCAGAA 3′ and *TDH3*-195-R 5′ GTCGTCACCAGAAGCAGTGA 3′. Expression of the *PHO4* gene was normalized to that of the housekeeping gene *TDH3*.

### Phosphatase assay

*C. albicans* strains from glycerol stocks were plated on YPD plates, grown overnight at 37°C, and few cells were used to inoculate liquid YPD. After overnight growth at 37°C, 200 rpm (C25 Incubator Shaker, New Brunswick Scientific), 1∶100 dilutions were performed in fresh YPD medium, and *C. albicans* cultures were allowed to grow under the same conditions to OD 2.0. Cells were spun down (5,000xg, 6 min) and resuspended at final OD600_nm_ = 0.5 in liquid PNMC medium containing 25 µM or 25 mM potassium phosphate buffer, pH 6.0. Cells were allowed to grow overnight and then supernatants were collected by centrifugation at 10,000xg for 5 min and used for the phosphatase assay using 6, 8-difluoro-4-methylumbeliferyl phosphate (DiFMUP, Molecular Probes) as substrate. Specifically, 50 µl of 0.1M sodium acetate, pH 4.2 was dropped in black, clear bottom 96 well plates (Corning Incorporated COSTAR) followed by the addition of 50 µl of supernatants (or fresh culture medium as background control) and 50 µl of 200 µM DiFMUP in 0.1M sodium acetate, pH4.2. Fluorescence was followed dynamically at an excitation/emission of 400±40/460±10 using a Microplate Fluorescence Reader FLx800 (Bio-TEK Instruments, Inc.).

### Statistical analysis

Statistical analysis of the data was performed with Student t-test using Sigma plot software and Kaplan-Meier survival curves using SPSS software.

## Supporting Information

Figure S1 **Colony morphotype in strains of** ***C. albicans*** **isolated from stool of critically ill patients.** (A) Microscopy images of *C. albicans* colonies grown on agarized PNMC-Pi↓ media. (B) Percentage of colonies observed to be filamentous on PNMC-Pi↓ and PNMC-Pi↑ agarized media.
(TIFF)
