# Supplementary figures and images for: Candida albicans Isolates from the Gut of Critically Ill Patients Respond to Phosphate Limitation by Expressing Filaments and a Lethal Phenotype
Source: PLoS One. 2012 Jan 13;7(1):e30119. doi: 10.1371/journal.pone.0030119 (PMC3258262; doi:10.1371/journal.pone.0030119)

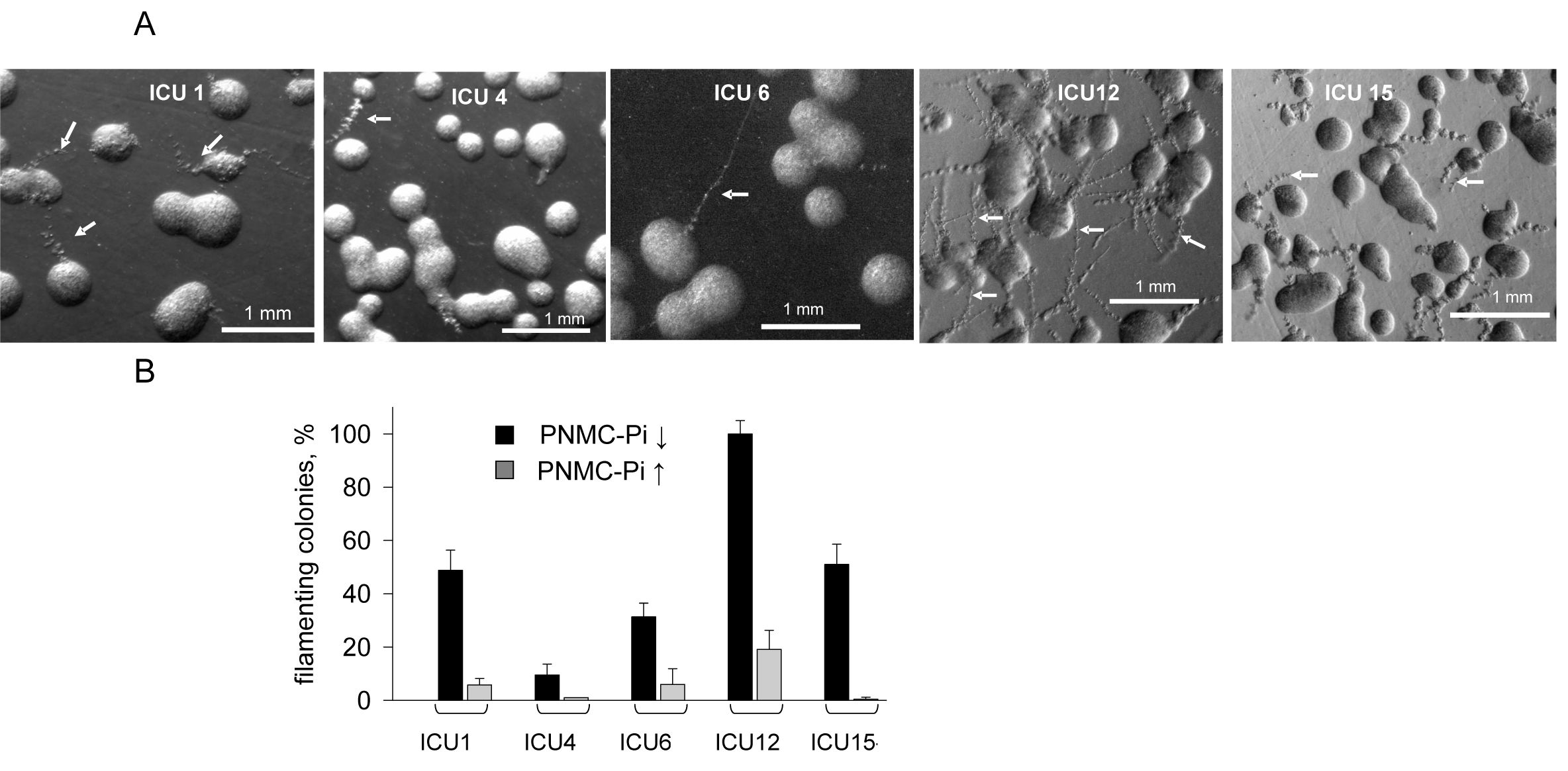

Supplement: Figure S1 — Colony morphotype in strains of C. albicans isolated from stool of critically ill patients. (A) Microscopy images of C. albicans colonies grown on agarized PNMC-Pi↓ media. (B) Percentage of colonies observed to be filamentous on PNMC-Pi↓ and PNMC-Pi↑ agarized media. (TIFF) [file pone.0030119.s001.tiff]
